# Application of Micro-Scale 3D Printing in Pharmaceutics

**DOI:** 10.3390/pharmaceutics11080390

**Published:** 2019-08-03

**Authors:** Andrew Kjar, Yu Huang

**Affiliations:** Department of Biological Engineering, Utah State University, Logan, UT 84322, USA

**Keywords:** additive manufacturing, 3D printing, drug delivery, micromedicine, drug development, micro-swimmer, micro-implant, oral dosages, microneedle, high-precision targeting, controlled release, geometry, resolution, feature size, personalized medicine, release profile, vascularization

## Abstract

3D printing, as one of the most rapidly-evolving fabrication technologies, has released a cascade of innovation in the last two decades. In the pharmaceutical field, the integration of 3D printing technology has offered unique advantages, especially at the micro-scale. When printed at a micro-scale, materials and devices can provide nuanced solutions to controlled release, minimally invasive delivery, high-precision targeting, biomimetic models for drug discovery and development, and future opportunities for personalized medicine. This review aims to cover the recent advances in this area. First, the 3D printing techniques are introduced with respect to the technical parameters and features that are uniquely related to each stage of pharmaceutical development. Then specific micro-sized pharmaceutical applications of 3D printing are summarized and grouped according to the provided benefits. Both advantages and challenges are discussed for each application. We believe that these technologies provide compelling future solutions for modern medicine, while challenges remain for scale-up and regulatory approval.

## 1. Introduction

Pharmaceutical development is commonly considered to proceed in three main stages: drug discovery, drug development, and drug delivery [1]. In drug discovery, a suitable target is first identified. Afterwards, a library of compounds is screened for activity with the biological target, from which an active compound is selected. In drug development, the active compound is then tested in various settings, including in vitro models, in vivo animal studies, and clinical trials. During these regulatory phases of intense development and testing, the exact dosing and delivery methods are optimized. Drug delivery is comprised of precise delivery of the active pharmaceutical compound and encompasses possibilities from oral dosages to drug-eluting micro-implants.

There are challenges which currently face each phase of pharmaceutical development. Drug development is a long, costly process. Typically, a new pharmaceutical entity will take over a decade to enter the market, requiring upwards of one billion dollars [2]. However, many drugs fail in later stages of clinical trials [3], as in vitro and animal models fail to fully predict drug reaction in humans. Animal models often produce data that are limited in their ability to translate to humans [4]. To this end, a heavy focus of drug development research seeks more realistic and effective in vitro models. 

Manufacturing better models for drug development proves to be challenging because the in vivo response is highly nuanced [5]. A key feature of human biological systems is exquisite spatial patterning and organization, down to the micron range It has long been known that cell response, morphology, chemotaxis, messaging, and differentiation depend on the micro-scale environmental conditions. Thus, in vitro models seek to faithfully recapitulate critical features of in vivo as closely as possible [6]. 

The goal of pharmaceutical drug delivery is similarly complex: the ideal drug should be highly specific in reaching and affecting its intended target, while minimizing side effects [7]. Many compounds display inhibitory or therapeutic effects at some minimum concentration, but increase in toxicity and side effects with increased dose, having some functional therapeutic window. Some drugs may have narrow therapeutic windows [8], need to be tissue-specific [9], or are specific to certain patients’ genetics. Individual biological systems are incredibly sensitive to location, dose, and timing of medication. Because these effects are sophisticated, medicine tailored to an individual is an attractive target for the industry [9]. 

However, today’s market has a limited possibility to produce personalized medicines [10]. For example, in the case of oral delivery, conventional batch methods cannot feasibly make every dose size. Splitting doses to overcome this is associated with dose variation, and it compromises dose coatings [11]. Additionally, current manufacturing cannot make different shaped tablets [10]. Inflexible dosage regimes highlight a need in the pharmaceutical industry that cannot be met through current manufacturing methods. Thus, innovative solutions are necessary. 

This review aims to cover recent advances in additive manufacturing with regards to micro-sized biomedical applications, and the potential solutions they provide to these stated challenges in drug delivery and development. The purpose of this article is to show that the integration of 3D printing technology has unique advantages. At a micro-scale, 3D-printed materials can provide nuanced solutions to controlled release, minimally invasive delivery, high-precision targeting, biomimetic models for drug discovery and development, and future opportunities for personalized medicine. Specific micro-sized pharmaceutical applications of 3D printing are summarized and grouped according to the provided benefits.

## 2. Additive Manufacturing: Methods and Resolution

**Figure 1:**
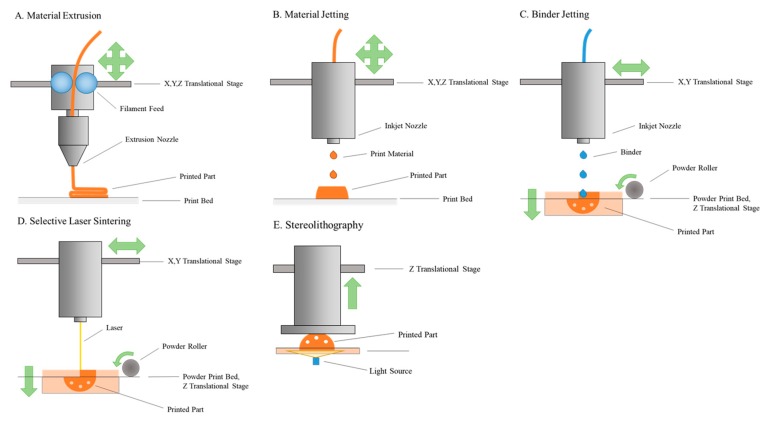
Typical additive manufacturing mechanisms. Additive manufacturing techniques are classified by their deposition of material in a layer-by-layer fashion. Material extrusion (**A**) traditionally deposits thermoplastic materials, but also includes pneumatic and mechanical deposition of semisolid materials. Both material jetting (**B**) and binder jetting (**C**) rely on familiar inkjet heads; in material jetting the entire print material passes through the nozzle, while in binder jetting, only a binder is deposited. An advantage of binder jetting is the support of the powder bed, negating the need for support structures or sacrificial material. This mechanism is also seen in selective laser sintering (**D**), where the powder bed is selectively fused by a laser. Finally, stereolithography (**E**) selectively polymerizes a liquid resin vat, thereby producing the desired part.

3D printing, more formally known as additive manufacturing, is rapidly becoming one of the most well-known and innovative technologies of the 21st century. Additive manufacturing is a number of manufacturing techniques in which material is selectively placed in a layer-by-layer fashion (Figure 1), including material extrusion, material jetting, binder jetting, selective laser sintering, and vat polymerization. Each of these techniques is currently applied in the pharmaceutical field, and thus are presented here [12,13,14,15,16,17,18,19,20,21,22,23,24,25,26,27,28,29,30,31,32,33,34,35]. 

### 2.1. Material Extrusion

Material extrusion is the least costly and most common type of 3D printing [36]. There are two major categories: fused deposition modeling and semisolid extrusion [37]. In both approaches, the preprinting material is extruded in a continuous stream through a nozzle. The nozzle or the platform (or a combination of the two) is moved in the x, y, and z directions to produce the final desired geometry. 

Fused deposition modeling utilizes a heated extrusion nozzle. Filaments are fed through the heated nozzle and then deposited onto the print bed containing the emerging part. As the filament cools, the layers fuse together. Heat-based printer nozzles are essentially limited to thermoplastics, as the material must decrease in solidity and viscosity as the temperature is increased, and then harden and bond on the print bed [38]. For pharmaceutical applications, filaments are usually prepared by incorporating active compounds via hot melt extrusion; however, recent advances include direct powder extrusion, which circumvents this process, and may enable a larger number of printable materials [39]. 

Semisolid extrusion techniques extend extrusion printing to a wider range of temperatures and materials, including living material (termed “bioprinting”) [40]. Instead of relying solely on heat, semisolid extrusion printers can print a variety of materials using pneumatic or mechanical extrusion forces. In these systems, the rheological properties of the fluid to be printed and the method of solidification must be carefully considered. For steady extrusion, fluids must have the correct viscosity. Non-Newtonian fluid behavior, such as shear rate dependence, is often a factor that must be taken into account [41]. After extrusion, solidification can occur through physical or chemical processes. For example, printers fitted with UV lamps can cross-link newly printed layers of photolinked hydrogels. Alternatively, the crosslinking agent may be printed at the same time as the print material [42]. These gelation processes are a key consideration in the design of materials to be used in semisolid extrusion prints.

The motions in the x–y plane and z-axis are typically actuated with extreme precision—down to 1 micron. In terms of feature resolution, fused deposition modeling is essentially limited by the size of the extrusion nozzle. Typical sizes of fused deposition nozzles are in the range of 400 microns [43,44,45,46]. Semisolid extrusion usually has relatively poor feature resolution, as lower-viscosity substances spread upon printing [37]. 

Hybrid techniques can push the feature resolution of extrusion printing to the range of ten microns [47]. In these printers, the extruded filament is subjected to an electric force, producing a much smaller filament stream. These “electrospinning” hybrids are commercially available and represent the highest resolution extrusion-based methods currently available [48,49].

### 2.2. Material Jetting

The mechanism of material jetting is similar to that of familiar inkjet printers. For 3D materials, the print head and platform are actuated to move in the x-, y-, and z-axis. For successful printing, the material must be cross-linkable upon delivery. As with semisolid extrusion, cross-linking processes include photo, thermal, ionic, and pH-dependent effects [41]. One of the greatest advantages of this technique is that it may be used to print multiple materials simultaneously, even materials with different properties [50]. Material jetting is used for both small molecules and bioprinting [40,41].

The final feature resolution of material-jetted prints depends on the droplet size. Print feature resolution is also highly dependent upon the rheological properties of the fluid and print speed, which must be carefully parametrized. Upon arrival onto the print, droplets tend to spread before they are fully cross-linked, which limits inkjet resolution. Additionally, the most defined features tend to be printed in parallel to the inkjet direction [36]. Inkjet manufacturers advertise feature resolution in the range of 20 to 100 microns [51].

### 2.3. Binder Jetting

While material extrusion and material jetting may be printed onto a variety of surfaces, binder jetting requires the use of a powder which may be selectively bound by the addition of a liquid binder. For the creation of each layer of the part, a layer of powder is spread across a printing surface [36]. An inkjet head then deposits binder in the desired geometry. The powder bed is then lowered, the new powder is spread, and another layer is selectively bound. This technique can require high volumes of powder, but has no need for sacrificial materials, as the powder bed can support the emerging part [52]. 

Advantages of this technique include the possibility of multimaterial printing, as multiple binding agents may be used [36]. Additionally, this manufacturing process can often occur at room temperature, and it easily makes porous structures [36]. Metals and ceramics are commonly used, but polymers may also be printed [36]. Materials printed in this way require postprocessing, such as chemical treatment, for better mechanical properties. Many printers have a feature resolution of two millimeters [36], but better printers can produce up to 50-micron feature resolution [12], dependent on powder particle size [36].

### 2.4. Selective Laser Sintering

Selective laser sintering occurs in a similar fashion to binder jetting, but instead of using a binder to fuse powder particles, a laser is used to sinter them together. Because of this mechanism, only materials that can be fused by laser are utilized. Metals and ceramics are common, although the use of thermoplastics is increasingly prevalent, especially in biomedical applications [13,53,54,55]. For all materials, careful calibration of the powder to be used is important, as particle size will affect feature print resolution, workability of particle spreading, and final print mechanical properties [56,57].

The feature resolution of selective laser sintering depends highly on the material. While some report feature resolutions in the range of 100 microns [12], selective laser sintering can be a high-resolution technique, producing features as small as 30 microns [58]. As with binder jetting, parts are usually porous and need postprocessing for smooth surfaces and mechanical strength. Additionally, selective laser sintering is usually fast and economical, requiring no support materials. When printing metals or ceramics, sterilization via an autoclave is a viable option.

### 2.5. Stereolithography

The oldest form of additive manufacturing, stereolithography, relies on the same techniques as its predecessor, photolithography. Stereolithography is based on the reaction of light with photopolymer resins. First, a large vat is filled with resin and subjected to a radiation source from the top or the bottom, in a desired geometric pattern. The bottom-up method, which features lower resin volumes, places the light source under the resin tank with a transparent base (as pictured in Figure 1) [37]. The light-cured layer polymerizes, and the build platform moves upwards, peeling the cured layer off the bottom surface. Another layer is then polymerized in a similar fashion. Alternatively, in top-down stereolithography, the actuator platform is lowered for each layer, requiring larger volumes of material for the part to remain fully immersed [59]. Continuous liquid interface processing (CLIP) and digital light processing (DLP) are other techniques related to stereolithography [37]. Stereolithographic techniques can offer relatively high resolution, by reaching the diffraction limit of light simply using conventional radiation sources. Some print quality can suffer from nonspecific photopolymerization due to light leakage [60]; however, the highest feature resolution is often reported as 20–30 microns in commercially available printers [61,62,63]. 

The printing process often requires post-curing, postprocessing, and sacrificial support structures. There is also a limited number of materials that may be used, but these include, prominently, photocurable polymers [37]. Some metals and ceramics may be printed in specialized machines and processes [64]. Recent studies have demonstrated the incorporation of active pharmaceuticals in the resin to be effective [65,66].

With increased cost, increased feature resolution is possible with two-photon polymerization, a specialized form of stereolithography. In two-photon polymerization, resins are polymerized using two laser beams. These machines can exceed the diffraction limit and produce feature resolution of 120 nm [67,68].

### 2.6. Resolution

**Figure 2:**
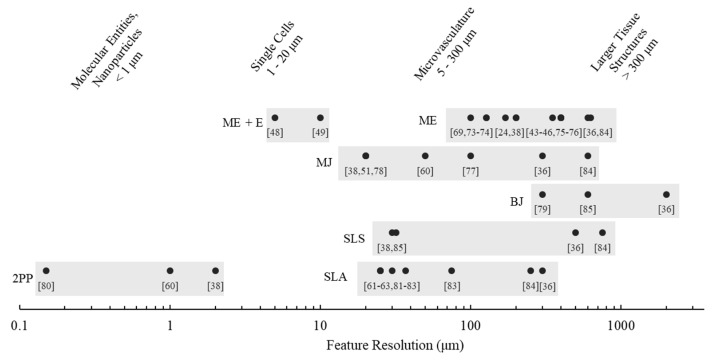
Maximum feature resolution of various 3D printing techniques, as compared to typical biological entities. Most techniques can print in the range of microvasculature; expensive, specialized methods, such as two-photon polymerization (2PP) and electrospinning hybrid extrusion (ME + E) are required for printing sizes comparable to single cells. Material extrusion (ME) feature resolution is essentially limited by nozzle size, while material jetting (MJ) and binder jetting (BJ) feature resolution is limited to droplet size. Binder jetting and selective laser sintering (SLS) feature resolution both depend on particle powder size, while stereolithography (SLA) has superior feature resolution based on the light source. However, for all print types, feature resolution depends highly on the designed geometry and print orientation. Data was compiled from manufacturer technical specification sheets [43,44,45,46,48,49,51,61,62,63,69,73,74,75,76,77,78,79,80,81,82,83] and literature [24,36,38,60,84,85]. Material extrusion values extracted from specification sheets are based on nozzle diameter.

The preceding feature resolutions are given as a range based on manufacturer specifications and literature (Figure 2 and Table 1). However, the reporting of resolution is non-standard across the industry. Manufacturers report any of the following specifications; x–y resolution, layer thickness, part accuracy, and nozzle size [43,69]. 

Typically, the layer thickness is the most straightforward specification to find, and values range as low as 5 microns, depending on the type of printing being used [12]. This same value may, however, be inaccurately reported as x–y resolution. Print x–y resolution is often a function of the material printing method: material extrusion rarely can provide structures finer than the nozzle, powder methods are limited by the powder size, and inkjet printers are limited by droplet size [12]. 

The smallest feature resolution, which is dependent on geometry, print speed, temperature, and material, is of the uppermost relevance to the biomedical designer. However, the smallest feature resolutions are not standardly reported. For example, material extrusion manufacturers commonly report accuracy to 100 microns using a 400-micron nozzle, indicating a difference between accuracy and smallest feature size [43,46]. Literature has attempted to address this [70,71], but more research in the area would be invaluable.

Continued innovation will push the boundaries of the current resolution limits, but standard printing techniques are within the range of critical biological entities [72]. All printer types can print in the micro-scale, which is defined as printing of features smaller than 1000 microns. Additive manufacturing brings unique capability to this field. While topography on a micro-scale can be created relying solely on material properties, additive manufacturing allows engineering design of specific micro geometries. As discussed in the following sections, printing on a micro-scale—within the range of single cells and microvasculature—can produce unique solutions for drug discovery, development, and delivery.

## 3. Controlled Release

**Figure 3:**
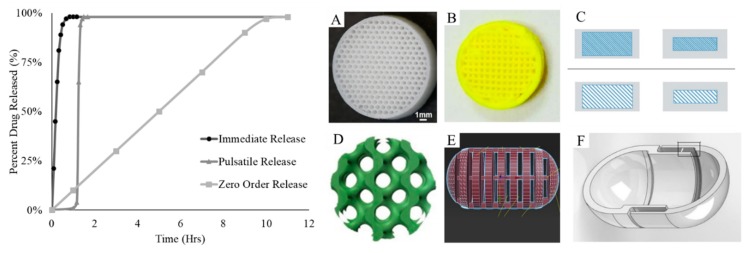
Idealized release profiles (left) and micro-geometry incorporation in oral dosages produced by additive manufacturing techniques (**A**–**F**). Immediate release is desirable for quick action drugs, such as pain relievers. Immediate release profiles are correlated to infill percentage (**A**) [91], and other factors such as wettability. Infill percentage may also be exploited for gastro-floating devices (**B**) [94]. If combined with a shell of variable thickness (**C**) [96], infill variation can also achieve tunable zero-order release. More complicated geometries offer release profiles that are dependent on erosion, providing immediate release profiles (**D**,**E**) [34,55]. Additionally, pulsatile release is possible with the fabrication of an outer shell of tunable thickness, here designed to be 600 microns (**F**) [33]. Reproduced with permission from Elsevier (**A**–**F**).

Additive manufacturing is gaining traction for use in drug delivery, having applications in drug delivery methods and devices [87,88,89]. Oral drug delivery devices, tablets, are one such application of additive manufacturing in the medical field, Spritam^®^ being the first FDA approved 3D-printed medicine. The incorporation of micro-geometry has unique advantages in terms of controlled release. Additive manufacturing can produce geometries that are impossible or impractical via typical pharmaceutical manufacturing processes (Figure 3).

One of the most straightforward geometric modifications—infill percentage—relies on the intrinsic material extrusion methods. Parts to be printed via material extrusion are commonly printed by first depositing an outer shell and subsequently filling this shell with preset infill geometry; 0% infill would leave the part fully hollow, while 100% infill creates a solid part. For use in oral drug delivery, Verstraete et al. demonstrated that lower infill percentages have faster release profiles [90]. Immediate release profiles are often desirable, as in pain relievers. Verstraete’s release profile results are due to the increase of surface area to volume ratio for the prints. Importantly, Kyobula et al. likewise demonstrated that this process is also dependent on wettability [91]. Spaces and cavities under the size of 600 microns were less wettable, producing longer release times than counterparts with >600-micron cavities. Other literature reports the correlation between infill, micro-geometry creation, and release profile [92,93]. Similarly, Li et al. showed that varied infill percentage could be tailored to create gastro-flotation tablets [94]. Prolonged retention enhances the bioavailability, and lower infill percentages produce floating without sacrificing mechanical properties such as friability. 

Conventional tablet release profiles are dominated by various physical forces, of which surface area to volume ratio plays a significant part. In these systems, drug release is often dominated by diffusion patterns. One method for creating more complex release profiles is the incorporation of outer layers, to produce, for example, enteric coatings which delay the release until the intestinal tract. These controlled release tablets are achievable through additive manufacturing, as demonstrated by Okwuosa et al., who showed that an outer coating of ≥520 microns was necessary to produce the intended release profile [95]. The feature resolution of the printer was also shown to affect the release profile, where low-resolution printing resulted in coating layers thicker than the nominal dimension.

For multilayer tablets, Zhang et al. demonstrated that release mechanisms are dependent on several parameters, including both infill percentage and shell thickness [96]. By varying these parameters, release profiles could be dominated by diffusion or swelling (or a combination), and the authors were capable of tuning until a zero-order release was achieved. Other literature similarly reports zero-order (or constant sustained) release [97,98]. These applications demonstrate the importance of small features in CAD designs for drug delivery. Micro-geometry and feature resolution have also been shown to be important in orodispersible, thin-layer films [99,100].

Other, less familiar shapes are also possible with additive manufacturing [35,101]. Whereas varied infill produces pores that are initially separated from the aqueous media, several groups have made channels and holes that cross the entire tablet. These channels were demonstrated by both Sadia et al. and Arafat et al. to produce much faster release profiles, putting them within the pharmacopeial regulations for immediate release [30,34]. These release profiles were a function of geometry; features in the range of 1000 microns seem optimal. Erosion becomes a dominant force in the dissolution of these tablets, as the tablet breaks into pieces as time progresses. Likewise, Fina et al. found faster dissolution for their gyroid structures, which could be paired with nonporous regions for complex release profiles [55].

Many of the presented examples rely on specific pairings of active pharmaceutical and polymer, which are then extruded through hot-melt extrusion [30,33,35,86,90,96,102]. The properties (rheology for material jetting, thermoplasticity for material extrusion, and particle size for binder jetting) must be carefully tuned and parameterized for each drug. Typically, active pharmaceutical is incorporated into filament at a rate of 4–8% *w*/*w* [31,35,55,91,94]. Some attempts have been made to create fully flexible systems. For example, Melocchi et al. have demonstrated a pulsatile release profile based on material extrusion of a shell (thickness 600 microns) which could be used for any number of active pharmaceuticals [33].

## 4. Minimally Invasive Delivery

Hypodermal needles are common for drug delivery in which oral ingestion is inappropriate; the method, however, is invasive. Additive manufacturing provides alternative solutions for minimally invasive delivery through the design of microneedle arrays.

The first transdermal drug delivery system was introduced in 1979, and since these systems have become more sophisticated with the addition of microneedle arrays [103]. Microneedle arrays, as compared to hypodermal needles, improve patient compliance, decrease pain and tissue damage, decrease the need for skilled healthcare professionals for administration, and inhibit microbial entrance [104,105]. Additionally, transdermally delivered drugs can elicit a higher immunogenic response and increased bioavailability. 

The efficacy of such systems is highly dependent on geometric properties. As Johnson et al. note, key parameters in the design of microneedle arrays include microneedle shape (height and diameter) aspect ratio, composition, strength, sharpness, spacing, and quantity [28]. For example, a decrease in aspect ratio corresponds to an increase in microneedle mechanical strength [106], whereas material composition and toughness facilitate penetration deepness [28]. Needle spacing is directly related to how much force is necessary for penetration [107]. Dimensions are variable, depending on the application. As Lu et al. note, various microneedle heights ranging from 150 to 2000 microns have been reported [103]. The microneedle array must at least penetrate the stratum corneum layer, the outermost layer of the skin, which is in the range of 10 to 20 microns [108]. To draw blood, the microneedle height must be at least 900 microns [109]. Optimal tip size is a function of material—robust materials have improved penetration at small tip sizes, while polymers at the same dimensions easily fracture [106]. The possibility of creating these structures via additive manufacturing is, therefore, limited by the feature resolution of the technology. Stereolithography (including two-photon polymerization) is the additive manufacturing technique most widely used for this purpose [22,27,28,104,109,110,111,112,113,114,115].

**Figure 4:**
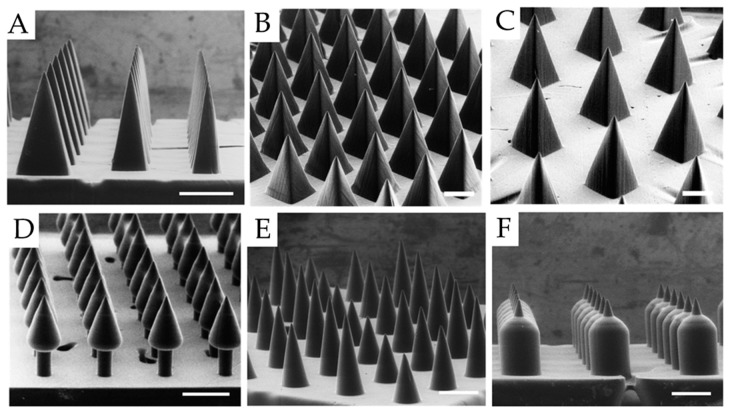
Microneedle providing minimally invasive delivery. Additive manufacturing brings enhanced flexibility, as shown in arbitrarily shaped microneedle arrays (**A**–**F**) [28]. Scale bars: 500 µm (**A**–**F**). Reproduced with permission from PLOS.

Different shapes are possible, each tailored for various applications (Figure 4). Pere et al. demonstrated that between cones and pyramids, cones took less force to penetrate, perhaps due to the decrease in microneedle-to-skin contact area [104]. Solid microneedles such as these are coated with active pharmaceutical ingredients, which are then deposited upon application. These systems must be carefully tuned for full biocompatibility and resistance to fracture. Another approach is to puncture the skin using solid microneedle arrays, and then apply drug topically, improving topical access via the punctures. Daraiswamy and Gittard developed various hollow microneedles with complex hollow geometry [111,115]. After puncture, an active pharmaceutical may be added, facilitating delivery. Alternatively, active pharmaceuticals may be incorporated with the needles and applied simultaneously. Needles are removed after application. 

Fully dissolvable microneedle arrays are a viable strategy for prolonged release. These arrays are typically made through polymer molding, which is not an additive manufacturing technique [116]. Limited print material capability in stereolithography and resolution limits in other methods challenge additive manufacturing for needles of this kind. As innovative materials are developed for stereolithography, and high-resolution versions of other printing types become available, this strategy may be realized. Currently, inkjet printing for coatings of microneedle arrays has received attention as a high-resolution, highly flexible method [104,105,116,117,118,119,120].

Luzuriaga et al. showed an innovative approach to the fabrication of microneedles, extending the technology to material extrusion [24]. However, as expected, the feature resolution of the printer resulted in the impossibility of creating sharp peaks. The smallest producible tip diameter was more than twice the optimal size, so postprocessing in basic solution was necessary to produce viable microneedles. This speaks to why stereolithographic techniques dominate these applications. It should be noted, however, that standard resolution stereolithographic printing reports distortion in final print features, compared to the CAD model [27]. Despite challenges, additive manufacturing of microneedle arrays can streamline prototyping and enable the fabrication of complex geometries [28].

## 5. High-Precision Targeting

**Figure 5:**
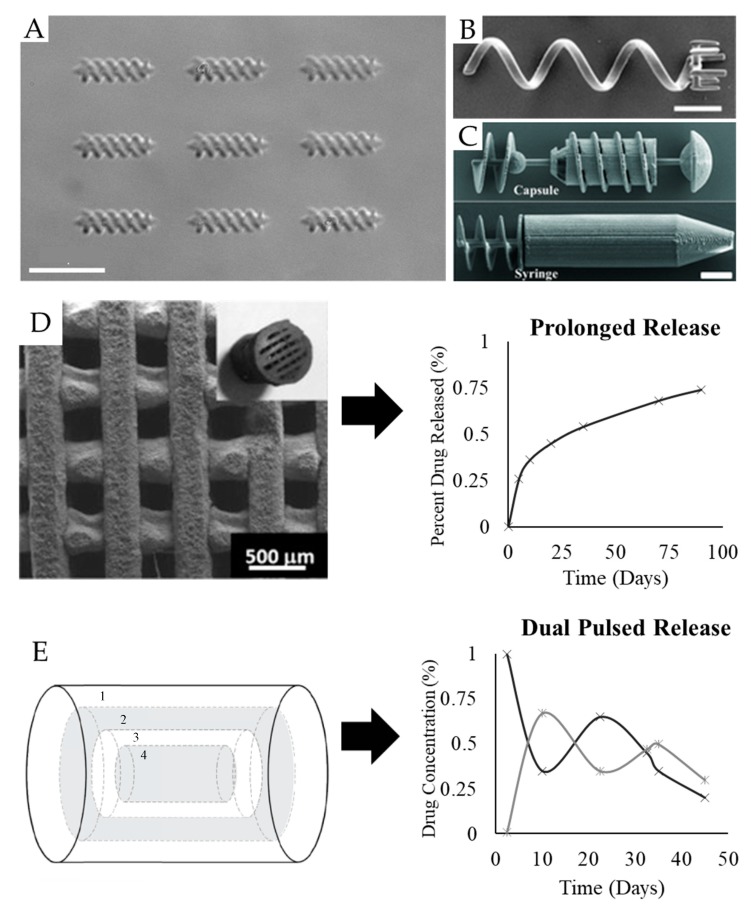
Examples of high-precision targeting in drug delivery. Micro-swimmer double helices (**A**) [121] are guided by a magnetic field to their target, being loaded during fabrication, while other micro-swimmers mechanically pick up the cargo (**B**) [122]. With high-resolution printing, complex micromachine structures can be fabricated, including capsules and syringes (**C**) [18]. Micro-implants can offer long term release profiles, based on drug incorporation within the scaffold in material extrusion (**D**) [123]. The dual-pulsed release is also possible, via geometric patterning in binder jetting (**E**) [124]. Scale bars: 20 µm (**A**,**C**) and 10 µm (**B**). (**A**) Reproduced with permission from American Chemical Society (**A**), Wiley (**B**,**C**), and Elsevier (**D**).

Some drug delivery applications require high-precision targeting, as do cancer treatments [7]. Highly specific delivery aims to provide a higher dose to a localized area while simultaneously reducing systemic toxicity. This delivery is often intended for parts of the body that are hard-to-reach and confined, thus making them difficult to approach through conventional methods. Thus, two lines of innovation—micro-swimmer devices and micro-implants—are fabricated to provide solutions (Figure 5).

### 5.1. Micro-Swimmer Devices

Micro-swimmers are motile delivery devices currently under developmental research. These devices are reviewed elsewhere [125,126]; the basic principles are summarized here to highlight the incorporation of additive manufacturing into the field. Micro-swimmer devices function on a variety of mechanisms, but all have essentially three stages: loading, transportation, and release. Micro-scale geometry can contribute significantly to each stage.

Loading can be achieved through passive adsorption [19,127], surface chemistry, incorporation of pharmaceuticals in the print material [23], or mechanical trapping within arms or syringes [18,122]. In the case of arms, the micro-swimmer needs to be maneuvered carefully to entrap the particle in a ring of extending rods (Figure 5B) [122]. In the case of syringes, Huang et al. designed micro-swimmers with Archimedean screw pumps, which were magnetically actuated (Figure 5C) [18]. By alternating the magnetic field, the pump could be selectively turned one way or another, producing fluid vortices sufficient for particle trapping. These latter approaches are complex and not fully efficient—incorporation of the desired particles within the object material may prove to be the superior method. These latter means are, however, fully dependent on geometry.

Transportation mechanisms are then engineering for motion to the target tissue. Actuation methods may be based on magnetic, thermal, chemical, electrostatic, or mechanical stimuli [18]. Upon excitation by one of these stimuli, micro-swimmers will move in the desired direction, depending on their shape, working their way through in vivo vascular systems. Of these methods, magnetic actuation is prominent, as magnetic fields are noninvasive and body tissue may be considered essentially nonmagnetic. Micro-swimmer devices using this mechanism are either plated with magnetic material [18,127] or have magnetic material incorporated [23,121], so the devices will respond to a magnetic gradient or a rotating magnetic field. A rotating magnetic field is preferable for its increased strength [23].

At the micro-scale, viscous forces dominate inertial forces, especially for small devices in body fluid, a non-Newtonian fluid [128]. Thus, the geometry must be carefully constructed for motion on this scale. Natural solutions to this problem include flagella and cilia, and these solutions have inspired many of the current micro-swimmer devices [20]. A typical shape is a cylinder encased by a helix or double helix. Ceylan et al. remark that double helices are more stable than a single helix, and carefully parameterized their helix to produce optimal swimming velocity [20]. Size is also important, as micro-swimmers are meant to maneuver easily within the target tissue. However, as Hunter et al. demonstrate, there is a trade-off between smaller size and higher possible velocity [23]. Typical helical structures have a length of 20 microns with a diameter of 5 microns. Other approaches to transport are not dependent on helical geometry, but instead on the incorporation of motile sperm cells [19]. Because these devices are currently in research development, testing of motion in vivo is limited. Similarly, testing in low-Reynolds regime fluids that accurately model body fluids is not consistent in all literature, a challenge which will need to be addressed for the application of these devices in pharmaceutical administration.

Release mechanisms vary and may be dependent on simple diffusion [23], light [121], magnetic fields [18], or mechanical stimuli [19]. Even structurally similar systems can be highly variable. One hydrogel system demonstrated enzymatic degradation to release the payload [20], whereas another used light as a trigger for tunable release [121]. Akin to oral dosages, release profiles are affected by erosion and swelling processes for hydrogel micro-swimmers or diffusion for non-hydrogel systems.

For these applications, high feature resolution is necessary. Thus two-photon polymerization is almost exclusively used. Two-photon polymerization is a cutting-edge technique for prototyping these devices: no other manufacturing methods parallel in shear flexibility. However, two-photon polymerization is essentially limited to photopolymers. Incorporation of particles (such as pharmaceuticals or magnets) in the resin is one strategy to achieve a wider variety of functional materials. Micromolding from 3D-printed molds has also been demonstrated [23], boasting a wider variety of possible materials.

### 5.2. Micro-Implants

Non-mobile drug delivery devices can also provide high-precision targeting and release. These drug delivery methods, termed micro-implants, can provide long-lasting release profiles, regenerative tissue effects, and restoration of tissue function. Implants are a long-standing part of conventional medicine; the use of additive manufacturing in implants is similarly well established [129]. Design considerations are important for both drug-eluting and inert implants. As shown here, additive manufacturing can enable solutions for both.

Microstructure is a key feature of implanted materials as it governs interactions with resident cells [130]. Optimal pore size, for example, is dependent on tissue type. Bone implants are made with pores in the range of 200 to 400 microns [131,132,133]. Pore size plays a role in differentiation, cell perfusion, and nutrient exchange, and stands as an example of important micro-geometry. 

Additive manufacturing has the unique capability of fabricating macrostructure and micro-geometry simultaneously. Most additive manufacturing techniques have been applied to making implant materials and tissue scaffolds, including material extrusion (both fused deposition modeling [21] and semisolid extrusion [29,134]), binder jetting [131,135], and selective laser sintering [14]. Because the microstructured design for many scaffolds is above 200 microns, the design of biomimetic pores is within the resolution range of most printers. 

Ideal bone implant materials should be biodegradable, osteoconductive, osteoinductive, angiogenic, and resistant to bacteria [132]. The incorporation and controlled release of compounds into the scaffold can help create these properties. Research has shown the incorporation of growth factors (esp. recombinant human bone morphogenetic proteins (rhBMP) or vascular endothelial growth factor (VGEF)) to enhance proliferation and bone response [29,134,136]. Antibiotics may also be feasibly incorporated [16,135]. 

Besides merely augmenting the properties of the scaffold, controlled release can provide pharmaceutical solutions (Figure 5). For example, in tuberculosis treatment, Zhu et al. and Wu et al. have demonstrated the incorporation of multiple drugs to provide programmed release [123,124]. While Zhu et al. showed prolonged release from a scaffold printing via material extrusion, Wu et al. designed dual-pulsed release by incorporating multiple layers of different drugs, manufacturing via binder jetting. In cancer treatment, Maher et al. showed biphasic release from their implant material [14]. This release is highly specific—providing stronger therapeutic effects and, importantly, lower systemic toxicity. While micro-swimmers must be guided to the target tissue, micro-implants are surgically placed in the area needing the most pharmaceutical treatment.

In these systems, the spatial distribution of the drug layers is a determining factor in the release profile, as was seen in oral dosages. For example, Martinez-Vazquez et al. showed first order kinetics due to their design [137]. Often, however, drug release profiles from scaffolds are biphasic: a quick release burst followed by prolonged release [14,131,134]. Prolonged release may be as long as 80 days [123]. Thus, there are clear benefits to the incorporation of additive manufacturing and pharmaceuticals into implants.

## 6. Biomimetic Models for Drug Discovery and Development

Whereas the preceding examples have all dealt with drug delivery, additive manufacturing can also be employed in the drug discovery and drug development phases. Perhaps the most viable application of additive manufacturing in drug development is the creation of organ models.

In manufacturing organ models, numerous techniques are currently employed. Monolayer cultures are an industry standard, due to their ease and reproducibility, despite the fact that the response of cells in such cultures is often different than in three-dimensional counterparts [138]. No methods have been able to produce fully biomimetic structures with the resolution and three-dimensional architecture found in vivo. However, fully functional organ models have the potential to provide better translational data towards clinical trials [6]. They also have a capacity to limit the cost later in the drug development process, excluding compounds earlier and increasing the accuracy of testing. This provides a strong impetus for development in this industry.

**Figure 6:**
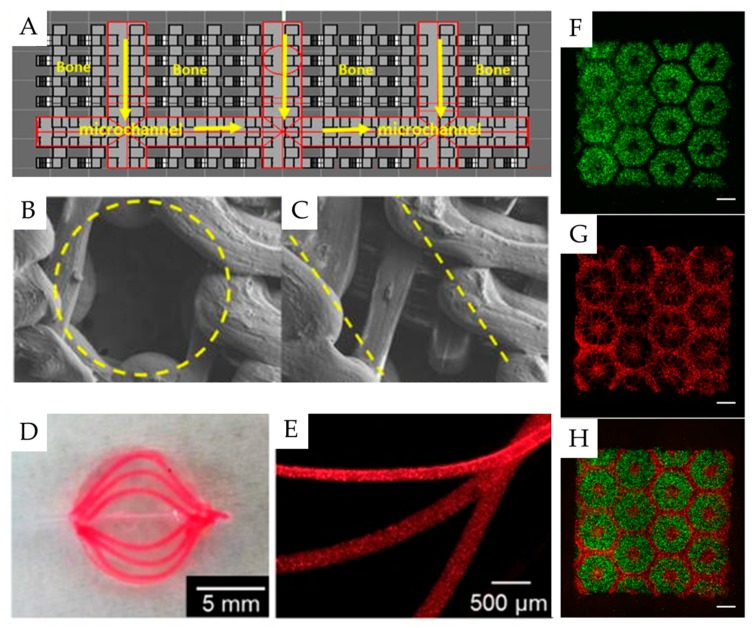
Examples of microstructured tissue constructs made via additive manufacturing techniques. Vascularization is a major component of functional tissue, and may be printed by leaving micron size spaces in the CAD file; 500-micron spaces are designed in this model (**A**–**C**) [15]. Another approach involves the printing of sacrificial material (**D**,**E**) [143]. Finally, tissues function depends on desired arrangement of cells, which may be achieved by direct bioprinting (**F**–**H**) [144]. Hepatic progenitor cells are marked in green, whereas support cells are marked in red. Scale bar: 500 µm. Vascularized, functional tissue provides better data for drug development. Reproduced with permission from Wiley (**A**–**C**), AIP (**D**,**E**), and PNAS (**F**–**H**).

Additive manufacturing for use in drug development has been extensively reviewed elsewhere [139]. Thus, we focus on demonstrating how micro-scale geometry is a key consideration in the design of organ models and functional tissue (Figure 6). 

Vascularization in vivo is a prime example of the importance of micro-scale geometry. Microvasculature is composed of arterioles, capillaries, and venules, which form a complex network [140]. In this network, lumen diameters range between 5 and 200 μm for capillaries and arterioles, respectively [140]. Without vascularization, the nutrient exchange is weak, and necrosis occurs. Much research, therefore, has sought to create vascularization, the realization of which would provide more fully biomimetic structures [141]. For pharmaceutical development, the incorporation of vasculature helps promote realistic cell viability and drug response [142,143]. 

Various strategies exist in the creation of microvasculature; additive manufacturing brings unique approaches. Printing methods include the incorporation of sacrificial materials, designed spaces, or even direct printing of endothelial cells (Figure 6A–E) [140]. Scaffolds printed with sacrificial materials or designed spaces are typically seeded after printing and postprocessing, while bioprinting is capable of placing cells throughout the scaffold. The defining feature of these systems is a need for simultaneous design of macro and micro features, which additive manufacturing is uniquely suited to create. However, capillary-sized features are beyond the current printer feature resolution for the majority of extrusion printing, the most common type of bioprinting [139]. Advances in the feature resolution for additive manufacturing are of importance for this aim.

Various tissues are the focus for bioprinting, including skin, liver, bone, cartilage, cardiac, and adipose tissue [6]. Of these, the liver is most important to pharmaceutical drug development, as many drugs fail clinical trials due to the detection of toxicity to the liver. Liver function depends on its microenvironment [5]. In vivo, functional liver tissue is made of both hepatocytes and supporting endodermal and mesodermal cells [144,145]. With this in mind, Ma et al. designed a bioprinted organ slice, which depends on the micron resolution placement of hepatic and supporting cells (Figure 6F–H) [144]. As in the case of vasculature, designs of this complexity are made possible through high-resolution additive manufacturing techniques. Multimaterial printing methods bring an increased spatial control unseen with other manufacturing methods [142].

Other cells that might be used in drug discovery display responses to micro-scale geometry: cardiomyocytes display alignment based on feature widths [146], and have been patterned in a filamentous matrix for drug discovery based on these effects [147]. As discussed previously, pore size plays a role in cell differentiation for bone cells and adipose-derived mesenchymal stem cells [148]. Thus, cell response to the engineered environment should be carefully tailored to produce the intended cell morphology and differentiation. Micro-scale, cell-size features are a key design parameter. The goal of these engineering systems is to provide the optimal cell response for use in drug development, allowing for data with better translational and predictive qualities. Besides the use of bioprinted scaffolds for application in drug discovery and organ-on-a-chip and microfluidic devices are also emerging as alternatives that may be created via additive manufacturing [149].

## 7. Future Challenges and Opportunities

The future is bright for the use of additive manufacturing in the pharmaceutical field; however, this future is not without obstacles. Traditional methods are more suited for mass-production than is additive manufacturing. Whereas injection molding becomes more cost-effective as the production rate rises, additive manufacturing of prints remains constant in cost per part [150]. While additive manufacturing significantly reduces prototyping time, it takes more time per part than traditional methods such as injection molding [52]. Increasing print speed is challenging, as there is generally a trade-off between feature resolution and print speed [60]. Higher feature resolution printing methods, such as stereolithography, selective laser sintering, or electrospinning hybrid extrusion have increased the cost for materials and a higher amount of energy necessary for processing [52]. Thus, additive manufacturing is historically well suited for rapid prototyping and design of devices, but not mass production. However, the features discussed previously are dependent on geometry only possible with additive manufacturing. Therefore, the development of additive manufacturing for mass-production is of great interest to the pharmaceutical industry.

Material science will continue to be a key field for additive manufacturing development. While the selection of materials for printing has grown exponentially in the last decade [41,151], materials specifications will continue to limit and inform the feasibility of additive manufacturing processes for specific applications. In consideration of materials, bioprinting of extracellular matrix material is poised for high impact in the field of 3D bioprinted scaffolds [152]. Improvements in material possibilities and printing methods may facilitate larger-scale solutions.

While this article has mainly focused on resolution, other important material properties include printability, mechanical properties, and drug loading capacity [41,153]. For example, bone implants should mimic in vivo bone stiffness, and research on micro-implants characterizes mechanical properties such as Young’s modulus [21], compressive strength [29], and yield stress [15]. These properties are dependent on printing method, geometry, and drug loading content. Mechanical properties are also important in oral dosages; as previously stated, microstructured oral dosages can be carefully designed for zero friability [30]. High-resolution printing is complex and requires geometric design, material selection, and printing parametrization to achieve biomedical solutions.

These technologies are making personalized medicine more feasible, as the customizability of additive manufacturing remains the most apparent benefit. The time necessary to print to identical shapes is comparable to the time necessary to print to customized shapes. Additive manufacturing already has a widespread application in the dental industry, where patient-specific parts are necessary [154]. In a similar way, patient-specific therapy remains a promising application of additive manufacturing in the pharmaceutical industry. Point-of-care fabrication of tailored medications is becoming increasingly possible [155]. Orphan medications, which cannot profitably be manufactured at a large scale, could be produced on an individual, small-batch level to reach the needs of patients [156]. Additive manufacturing is uniquely suited for this application. 

In the case of oral drug delivery, various authors have addressed the printing of tablets with fully customizable release profiles [157]. As presented, these release profiles are tuned by infill percentage or geometric structure. For example, dose combination or “polypills” are an emerging possibility afforded by additive manufacturing [86]. Maroni et al. demonstrated a shelled capsule capable of dual-pulse controlled release [32]. Their design took advantage of wall thickness and polymer selection for the timing of release. Khaled et al. showed both a three-in-one combination and a five-in-one combination based on spatial separation of active ingredients [25,26]. For each of the preceding cases, the microstructure is a key feature enabled by additive manufacturing.

Larger features are also personalized. Lim et al. designed a finger splint which could be 3D-printed in tandem with microneedles for drug delivery [27]. The design hoped to optimize skin-to-microneedle contact, thereby increasing efficiency, being made specifically for the user’s hand. Drug releasing implants made via additive manufacturing also show macrostructure easily tailored to each patient [123]. Printed pediatric stints have already been shown to be effective personalized medical implants in hospital settings [158]. High-resolution printing, therefore, augments current efforts towards personalized medicine. These tablets could be designed, fabricated, and distributed on a case-by-case basis, the entire process occurring at the local clinic.

Regulation of these applications will likely prove to be one of the most challenging hurdles before the wide-spread application of this technology [159]. However, the implementation of microstructured devices made via additive manufacturing promises to shift the paradigm of the industry and enable solutions to the challenging and nuanced problems currently faced.

## Figures and Tables

**Table 1:** Representative commercial printers with their associated feature resolutions and applications in the literature. Feature resolution data is taken from manufacturer technical specification sheets. Material extrusion values extracted from specification sheets are based on nozzle diameter. However, the smallest feature size is dependent on geometry, print speed, temperature, and material, and is not standardly reported. High-resolution printing techniques find applications in the printing of oral dosages, microneedles, micro-swimmers, and micro-implants. Material extrusion is a popular technique in the printing of oral dosages, whereas the feature resolution of stereolithography and two-photon polymerization are necessary for use in microneedles and micro-swimmers.

3D-Printing Type	Printer	Resolution (µm)	Applications
Electrospinning Hybrid Extrusion	RegenHU Benchtop 3D Discovery Evolution	5 [48]	Oral Dosages [25,26]
	GeSiM Bioscaffolder 3.2/4.2	10 [49]	Micro-implants [29]
Material Extrusion	LulzBot TAZ 5	350 [75]	Microneedles [24]
	MakerBot Replicator 2, ZMorph 2.0 SX	400 [43,44,45]	Oral Dosages [30,31,32,33,34,35,86]
	Solidoodle 2 Base	400 [46]	Micro-implants [21]
Material Jetting	3D Systems Phenix PXM	20 [51]	Micro-implants [14,15]
Binder Jetting	Z Corporation Spectrum Z510	300 [79]	Micro-implants [16]
Two-Photon Polymerization	NanoScribe Photonic Professional	0.15 [80]	Microneedles [22] Micro-swimmers [19,20]
Stereolithography	Envisiontec Perfactory DSP III Standard SXGA+, Kudo 3D Titan 1, Carbon M2	25–75 [61,63,83]	Microneedles [17,27,28]
	3D Systems ProJet 6000	25 [62]	Micro-swimmers [23]
